# Spinal glial cell derived extra-pituitary prolactin contributes to postoperative pain in females

**DOI:** 10.3389/fnagi.2026.1741102

**Published:** 2026-02-03

**Authors:** Mayur J. Patil, Sergei Belugin, Michael Henry, Anahit H. Hovhannisyan, Priscilla A. Barba-Escobedo, Jennifer M. Mecklenburg, Vincent Goffin, Gregory Dussor, Theodore J. Price, Armen N. Akopian

**Affiliations:** 1 Department of Endodontics, University of Texas Health Science Center at San Antonio, San Antonio, TX, United States; 2 Department of Molecular Pharmacology & Physiology, University South Florida (USF), Tampa, FL, United States; 3 Inserm U1151/CNRS UMR8253, Université de Paris, Paris, France; 4 Department of Neuroscience and Center for Advanced Pain Studies, School of Behavioral and Brain Sciences, University of Texas at Dallas, Richardson, TX, United States; 5 Department of Physiology, University of Texas Health Science Center at San Antonio, San Antonio, TX, United States

**Keywords:** extra-pituitary, inflammatory pain, postoperative pain, prolactin, prolactin receptor, sex-dependent, spinal astrocytes

## Abstract

Peripheral and spinal prolactin (PRL) receptor (PRLR) signaling contributes to the female-selective regulation of pain. This study investigated the relative roles of pituitary-derived PRL (PRL_pit_) and extra-pituitary PRL (PRL_ext_) in these effects. Using STAT5 phosphorylation (pSTAT5) as a surrogate marker of PRL-responsive cells, we found that hindpaw incision-induced pSTAT5 in dorsal root ganglion (DRG) neurons depends primarily on PRL_ext_. Immunohistochemistry (IHC) revealed incision-triggered induction of PRL_ext_ in rodent female myelinated peripheral nerves, the epidermis, medium-to-large DRG neurons, and a subset of spinal astrocytes, some of which co-expressed the glial glutamate transporter GLAST. PRL_pit_ plays critical role in activation of PRLR during stress-induced pain conditions. However, blockade of PRL_pit_ by hypophysectomy or bromocriptine did not substantially alter incision- or IL-6–induced heat or mechanical hypersensitivity. In contrast, the PRLR antagonist Δ1–9-G129R-hPRL (ΔPRL) reduced pSTAT5 in DRG neurons and reversed postoperative hypersensitivity in females. Postnatal ablation of GLAST^+^ cells in GLAST^cre−ER/−^/DTA^fl/−^ mice attenuated incision-induced hypersensitivity in females but not in males, and ΔPRL had no additional effect in these mice, indicating that spinal GLAST^+^ astrocytes are a major source of pain-promoting PRL_ext_ in female rodents. These results demonstrate that extra-pituitary PRL, particularly from spinal GLAST^+^ astrocytes, is a key contributor to female-selective regulation of postoperative and inflammatory pain.

## 1 Introduction

Prolactin (PRL) has a wide range of physiological and pathophysiological functions (Ben-Jonathan et al., 2008). Understanding the mechanisms that regulate PRL production and release from both the pituitary and extra-pituitary tissues is critical for elucidating its diverse roles. The primary endogenous source of PRL is the pituitary lactotroph, a specialized cell type that secretes PRL in response to physiological demands. Pituitary-derived PRL (PRL_pit_) release is tightly controlled by a negative feedback loop: PRL stimulates dopamine release from hypothalamic tuberoinfundibular (TIDA) neurons, and this dopamine, in turn, inhibits PRL secretion from the pituitary (Demaria et al., 2000; Lyons et al., 2012).

A seminal report by Nagy and Berczi (1991) suggested the existence of extra-pituitary sources of PRL (PRL_ext_). In primates and humans, PRL_ext_ and PRL_pit_ are driven by distinct gene promoters (Hiraoka et al., 1991; Berwaer et al., 1994; Marano and Ben-Jonathan, 2014). In contrast, basal PRL expression in rodents is largely accounted by PRL_pit_ (Yip et al., 2012). However, injury or pathological conditions can induce PRL_ext_ in multiple rodent tissues (Marano and Ben-Jonathan, 2014). For example, inflammation or hindpaw incision surgery markedly upregulates PRL_ext_ at the injury site and within the spinal cord (Scotland et al., 2011; Patil et al., 2013a). Moreover, PRL_ext_ production is sex-dependent. Thus, while incision-induced PRL_ext_ levels are comparable between males and females at the injury site, they are significantly higher in the female spinal cord (Patil et al., 2013a). Induced PRL_ext_ can act as a cytokine (Montgomery, 2001; Langan et al., 2010), a local neurotransmitter, or a neuro-modulator (Torner et al., 2004). Despite extensive evidence for its production and actions, the physiological and pathophysiological roles of PRL_ext_ remain poorly understood across different conditions and diseases.

Peripheral prolactin receptor (PRLR) signaling regulates sensory neuron sensitization (Diogenes et al., 2006; Patil et al., 2013b, 2014; Liu et al., 2016) and contributes to injury-induced hypersensitivity in a female-selective manner (Patil et al., 2013a, 2019a; Chen et al., 2020a,b; Avona et al., 2021). This sex-specific effect is attributed to the female-specific functional expression of PRLR and *Prlr* gene in sensory neurons (Patil et al., 2019b; Avona et al., 2021). The rationale for the present study stems from the observation that PRL_ext_ levels increase under pain-relevant conditions, yet the role of PRL_ext_ in nociceptive transmission remains largely unknown. Therefore, this study aimed to determine the relative contributions of PRL_pit_ and PRL_ext_ to postoperative pain in female rodents, identify the cell types responsible for PRL_ext_ production and release and elucidate how PRL_ext_ acts on nociceptors to promote pain in a female-specific manner. A better understanding of the sources and mechanisms of PRL_ext_ action may enable the development of targeted therapies that alleviate pain without interfering with the essential endocrine functions of PRL_pit_, thereby minimizing potential side effects.

## 2 Materials and methods

### 2.1 Ethical approval

This study was conducted in accordance with the ARRIVE 2.0 guidelines (Percie du Sert et al., 2020). All animal care and experimental procedures complied with the U.S. Public Health Service Policy on the Use of Laboratory Animals, as well as ethical standards set by the National Institutes of Health (NIH) and the Society for Neuroscience (SfN), with a commitment to minimizing both animal use and suffering. Mice were euthanized via transcardiac perfusion following intramuscular injection of 100 μL of a 1:1 Ketamine/Dexdormitor cocktail. This method, recommended by the AVMA Guidelines for the Euthanasia of Animals, was chosen to ensure minimal distress. Mice and rats were housed under a 12-h light/dark cycle (lights on at 7:00 AM) with food and water available *ad libitum* in their home cages. All animal experiments were conducted in accordance with protocols approved by the Institutional Animal Care and Use Committees (IACUC) of the University of Texas Health Science Center at San Antonio (UTHSCSA) and the University of Texas at Dallas (UTD).

### 2.2 Animals and key reagents

Experiments were conducted on wild-type adult female and male C57BL/6 mice (3–5 months old) and Sprague-Dawley rats (200–250 g). Mice were obtained from The Jackson Laboratory (Bar Harbor, ME), and rats were obtained from Charles River Laboratories (Wilmington, MA). Hypophysectomized female rats were also purchased from Charles River Laboratories.

The GLAST^cre/+−ER^ mouse line (B6.129 background) was kindly provided by Dr. Martin Paukert (University of Texas Health Science Center at San Antonio, TX). The ROSA26^eGFP − DTA^ (Stock No. 006331), Ai14-RCL-TdT (Stock No: 007914), and RCL-ChR2(H134R)/EYFP (Stock No: 012569) mouse lines on B6.129 backgrounds were purchased from the Jackson Laboratory (Bar Harbor, ME). The PRLR antagonist Δ1-9-G129R-hPRL (ΔPRL) (Rouet et al., 2010) is a modified human PRL protein that binds to and blocks PRLR function in rats, mice, and humans (Bernichtein et al., 2003). The specificity of ΔPRL has been extensively validated *in vitro* (Bernichtein et al., 2003; Scotland et al., 2011) and *in vivo* (Rouet et al., 2010), including in *Prlr* knockout mice (Belugin et al., 2013). Bromocriptine mesylate was obtained from Tocris Bioscience (Minneapolis, MN). Estradiol (E2; Cat. PHR1353), tamoxifen (Cat. T5648-1G), and mouse IL-6 (Cat. IL017) were purchased from Millipore-Sigma (St. Louis, MO).

### 2.3 Procedures

The estrous phase was determined by vaginal lavage as previously described (Caligioni, 2009). Estrogen supplementation was achieved by intraperitoneal (i.p.) injection of estradiol (E2; 5 mg/ml in sesame oil, 60 μl) administered twice weekly for 3 weeks. To induce Cre-mediated recombination, 5-week-old mice received a total of four i. p. injections of tamoxifen (2 mg in 100 μl corn oil), administered every other day over 8 days. Tamoxifen-treated mice were used for experiments 4–6 weeks after induction. Pituitary PRL release was inhibited by three consecutive daily i.p. injections of bromocriptine mesylate (2 mg/kg in 100 μl corn oil containing 10% ethanol). Protocols for PRLR inhibition using Δ1-9-G129R-hPRL (ΔPRL) are detailed in the corresponding experimental sections. Inflammatory pain was induced by a single intraplantar (i.pl.) injection of IL-6 (1 ng). Hindpaw incision surgery was performed as previously described for mice (Pogatzki and Raja, 2003) and rats (Brennan et al., 1996). Sham-operated control mice underwent anesthesia, antiseptic preparation, and application of topical antibiotics without an incision (Brennan et al., 1996; Pogatzki and Raja, 2003).

### 2.4 Immunohistochemistry (IHC)

L3–L5 DRG, spinal cord corresponding to L3–L5 levels, and hindpaws from paraformaldehyde perfused rodents were fixed additionally with 4% paraformaldehyde, cryoprotected with 10% and then 30% sucrose in phosphate buffer, embedded in Neg-50^TM^ (Richard Allan Scientific, Kalamazoo, MI), and 20-30 μm cryo-sections (depending on tissue) were generated. To retrieve activated signal transducer and activator of transcription 5 (pSTAT5) antigen sites, glass slides with tissue sections were heated for 10 min at 90 °C in 0.01 M Tris, pH 10 (Sapsford et al., 2012). IHC was carried out as previously described (Belugin et al., 2013). The following antibodies were used in the study: PRL protein in rats was detected with rabbit anti-PRL (Agilent-DAKO, Santa Clara, CA; cat: A0569; 1:100) (Patil et al., 2014); PRL in mice was detected with rabbit anti-PRL (Bioss, Boston, MA; cat: BS-23763R; 1:200); pSTAT5 in rats was detected with rabbit anti-pSTAT5 (Tyr694, Cell Signaling Technology, Beverly, MA; cat: 9314S; 1:200) (Sapsford et al., 2012); rat DRG and spinal cord neurons were detected with mouse monoclonal anti-NeuN antibodies (Millipore-Sigma, St. Louis, MO; catalog MAB377; 1:100); mouse spinal astrocytes and Schwann cells in hind paw were labeled with chicken anti-GFAP (Neuromics, Edina, MN; catalog CH22102; 1:100); and rat and mouse monocytes/macrophages/dendritic cells/microglia were labeled with mouse monoclonal antibody OX-42 (CD11b/c; Bio-Rad, Hercules, CA; catalog MCA275GA; 1:50) (Alizadeh et al., 2017). Sections were incubated with species-appropriate donkey Alexa Fluor secondary antibodies (1:200; Jackson Immuno-Research, West Grove, PA). Images were acquired using a Keyence BZ-X810 All-in-One Fluorescent Microscope (Keyence, Itasca, IL) or a Nikon Eclipse 90i microscope (Melville, NY, USA) equipped with a C1si laser scanning confocal imaging system. Images were processed with NIS-elements software (Nikon Instruments, Melville, NY) and Adobe Photoshop PS (www.adobe.com). Control IHC was performed on tissue sections processed as described but either lacking primary antibodies or lacking primary and secondary antibodies. Z-stack IHC images were obtained from 3 to 5 independent tissue sections from 3 to 4 animals. Size of cells was measured using NIS-elements or Keyence software. To count numbers of DRG and spinal neurons, sections were labeled with NeuN. Number of labeled cells per captured image was counted using Image J. Intensities of labeled areas were assessed using Image J software (https://imagej.net/ij/download.html) as described (https://theolb.readthedocs.io/en/latest/imaging/measuring-cell-fluorescence-using-imagej.html). Subtractions of background intensity from signal levels were applied. Intensities of labeled areas (aka “mean gray values”) were normalized by area size.

### 2.5 Behavior experiments

Vehicle or drugs were administered via intraplantar (i.pl.) injection into the hindpaw and/or intrathecal (i.t.) injection into the spinal cord. The vehicle for ΔPRL was 0.9% saline. All behavioral experiments were blinded, with the experimenter unaware of treatment conditions. Animals were randomly assigned to experimental groups, and testing was conducted in small cohorts.

Heat hypersensitivity was assessed using a Hargreaves' apparatus (Ugo Basile). Animals were habituated to the testing environment for at least 1 h prior to testing and placed on a glass surface maintained at ≈20 °C. Thermal withdrawal latencies were measured in response to a radiant heat beam at each time point, with three measurements per animal averaged to generate the data point. To prevent tissue injury, the heat stimulus was terminated after 20 s if the animal did not withdraw its paw.

Mechanical hypersensitivity was evaluated using two methods. First, animals were habituated for 45–60 min, and baseline measurements were recorded from the right hind paw using a Dynamic Plantar Aesthesiometer (Ugo Basile) as previously described (Patil et al., 2013a). The device applies a gradually increasing mechanical force (0–50 g over 10 s) to the paw, and the withdrawal threshold is recorded in grams. Second, in mice, mechanical sensitivity was also assessed using von Frey filaments and the up-down method (Chaplan et al., 1994).

### 2.6 Statistics

GraphPad Prism 10.0 (GraphPad, La Jolla, CA) was used for all statistical analysis. Data are shown as mean ± standard error of the mean (SEM), with “*n*” referring to the number of animals for IHC and the number of independent animals per group in behavioral experiments. Differences between groups were assessed by chi-square analysis with Fisher's exact test, unpaired *t*-test, 1-way ANOVA with Bonferroni's *post-hoc* tests (each column was compared to all other columns), or 2-way ANOVA with multiple comparisons “compare cell means regardless of rows and columns” with Bonferroni's *post-hoc* test. Differences were accepted as statistically significant when *p* < 0.05. Interaction *F* ratios and the associated *p* values are reported in the text.

## 3 Results

### 3.1 Contribution of PRL_*pit*_ and PRL_*ext*_ to incision surgery-induced STAT5 phosphorylation in female rat DRG and spinal cord

Previous studies have shown that PRL, PRLR, and *Prlr* gene expression patterns and signaling are nearly identical in the hind paw, dorsal root ganglion (DRG), and spinal cord of rats and mice (Diogenes et al., 2006; Patil et al., 2014, 2019a,b). Experiments in this study were conducted in rats or mice, depending on the availability of reliable tools and transgenic lines. Using both species strengthens the findings, as physiological effects were consistent across rodents. Activation of receptors for cytokines, growth factors, and hormones leads to phosphorylation of signal transducer and activator of transcription 5 (pSTAT5) (Furigo et al., 2016; Rani and Murphy, 2016). Although pSTAT5 is not exclusively a marker for PRLR activation, multiple studies have demonstrated that PRL-induced pSTAT5 serves as a functional readout of PRLR signaling in neurons (Brown et al., 2010; Sapsford et al., 2012). For example, systemic treatment with bromocriptine mesylate, a dopamine receptor agonist (de Leeuw van Weenen et al., 2010), suppresses pituitary PRL_pit_ production and nearly eliminates basal hypothalamic pSTAT5 (Yip et al., 2012).

**Figure 1:**
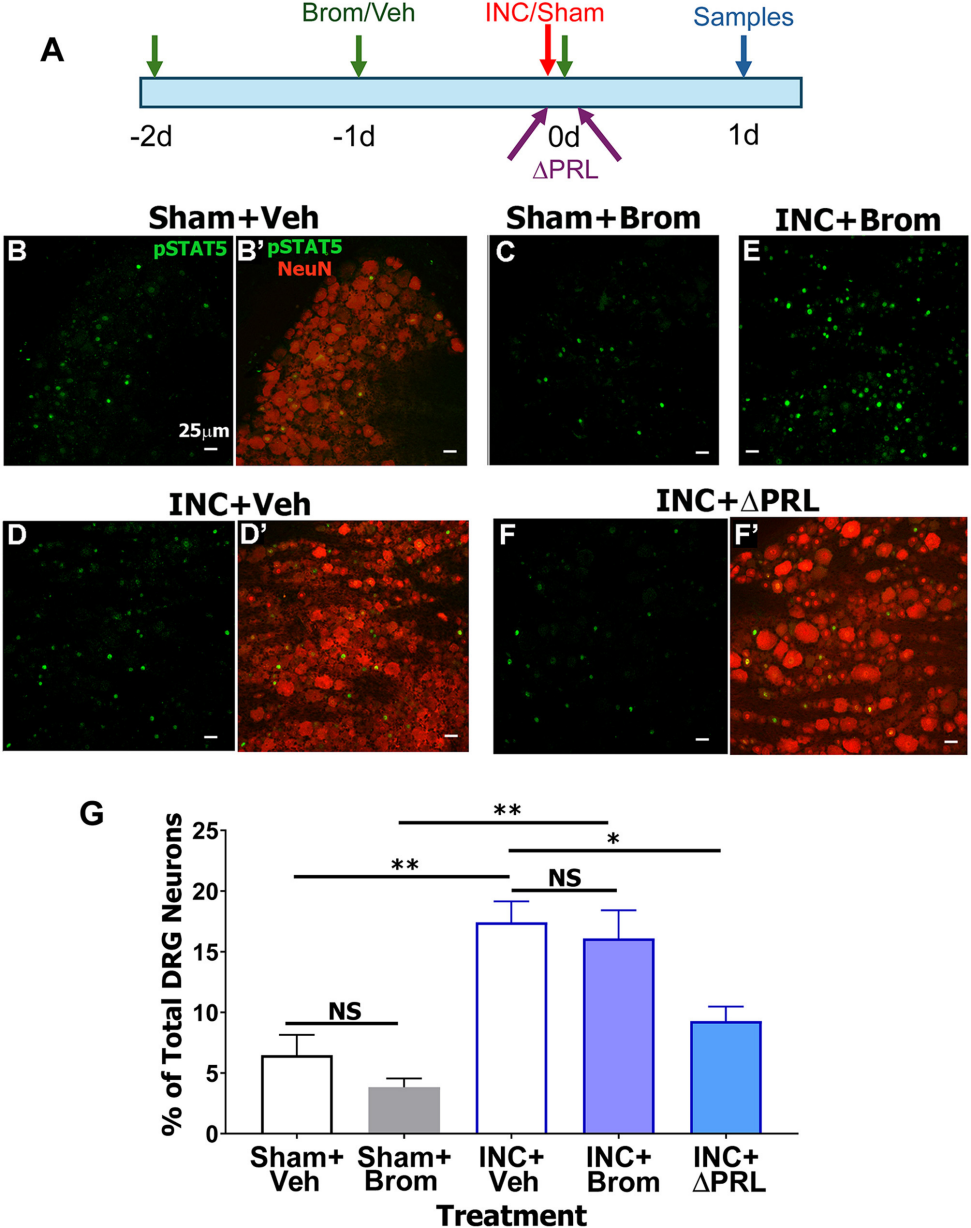
Effect of incision surgery, bromocriptine, and Prlrantagonist (ΔPRL) treatments on phosphorylation of STAT5 (pSTAT5) in female rat DRG neurons. **(A)** Schematic of experiments. Female rats received 3 consecutive i.p. treatments by vehicle or bromocriptine. Then, sham or incision surgery was performed. A group of female rats received ΔPRL (PRLR antagonist) injections both i.pl. and i.t. (5 μg per site) immediately after surgery, and again at 3 and 6 H post-incision. Samples were collected 1d after sham/incision procedures. L4–L5 dorsal root ganglion (DRG) sections from female rats were labeled with pSTAT5 and NeuN. NeuN immunostaining was used to quantify the total number of DRG neurons (349 ± 19 neurons; *n* = 36 sections). Representative immunohistochemistry (IHC) images of ipsilateral DRG are shown: **(B, B′)** pSTAT5 **(B)** and pSTAT5 + NeuN **(B′)** labeling from rats treated intraperitoneally (i.p.) with vehicle (Veh; saline) for 3 days and collected 1 day after sham surgery (Sham). **(C)** pSTAT5 labeling from rats treated with bromocriptine (Brom; i.p. for 3 days) and collected 1 day after sham surgery. **(D, D′)** pSTAT5 **(D)** and pSTAT5 + NeuN **(D′)** labeling from vehicle-treated rats collected 1 day after incision (INC). **(E)** pSTAT5 labeling from rats treated with bromocriptine (3 days, i.p.) prior to incision and collected 1 day post-surgery. **(F, F′)** pSTAT5 **(F)** and pSTAT5 + NeuN **(F′)** labeling from rats treated with the Prlr antagonist ΔPRL (5 μg per site, intraplantar [i.pl.] + intrathecal [i.t.]) immediately after incision, followed by additional ΔPRL injections at 3 h and 6 h post-surgery. **(G)** Quantification of pSTAT5′ DRG neurons (percentage of total ipsilateral DRG neurons) across treatment groups indicated on the X-axis. Data are presented as mean ± SEM; ^*^p < 0.05, ^**^p < 0.01; NS, not significant (one-way ANOVA; *n* = 3). Fluorescent micrographs were captured using a 20 × objective. Scale bar: 25 μm.

Female rats underwent sham or incision surgeries as previously described (Brennan et al., 1996) and received 3 consecutive days of i.p. vehicle or bromocriptine treatment prior to surgeries (Figure 1A) (Mason et al., 2022). pSTAT5 immunohistochemistry (IHC) was used as a surrogate marker for PRL responsiveness, while NeuN labeling allowed counting of total neurons in the DRG and spinal cord. Unlike non-phosphorylated STAT5, pSTAT5 is predominantly localized to the nucleus (Sapsford et al., 2012; Yip et al., 2012) (Figures 1B–F′). Basal pSTAT5 was observed in DRG of sham-operated, vehicle-treated female rats (Figures 1B, B′, G). Bromocriptine treatment in sham-operated rats did not reduce the number of pSTAT5^+^ neurons *(*Figures 1C–G). In contrast, incision surgery significantly increased the number of pSTAT5^+^ neurons in ipsilateral DRG of vehicle-treated rats (Figures 1D–G). Pretreatment with bromocriptine, which eliminates (or substantially reduces) PRL_pit_ (Mason et al., 2022), did not significantly reduce incision-induced pSTAT5^+^ neurons compared to vehicle-treated incision rats (INC+Veh vs. INC+Brom; 1-way ANOVA, Bonferroni *post-hoc* test; [*F*_(4, 10)_ = 13.6; *P* = 0.0005; *n* = 3; Figures 1D–G)]. These results suggest two possibilities: (1) surgery-induced pSTAT5 increases are driven by non-PRL inflammatory mediators unaffected by bromocriptine, or (2) incision induces peripheral and/or spinal PRL_ext_, which activates STAT5 via PRLR on sensory neurons (Patil et al., 2013a). To test this, female rats received ΔPRL (PRLR antagonist) injections both i.pl. and i.t. (5 μg per site) immediately after surgery, and again at 3 and 6 H post-incision (Figure 1A). DRG collected 1 day post-incision showed that ΔPRL substantially reduced incision-induced pSTAT5^+^ neurons (Figures 1D, D′ **vs**. 1F, F′, G), indicating that PRL_ext_ likely contributes to incision-induced pSTAT5 activation in female rat DRG neurons.

**Figure 2:**
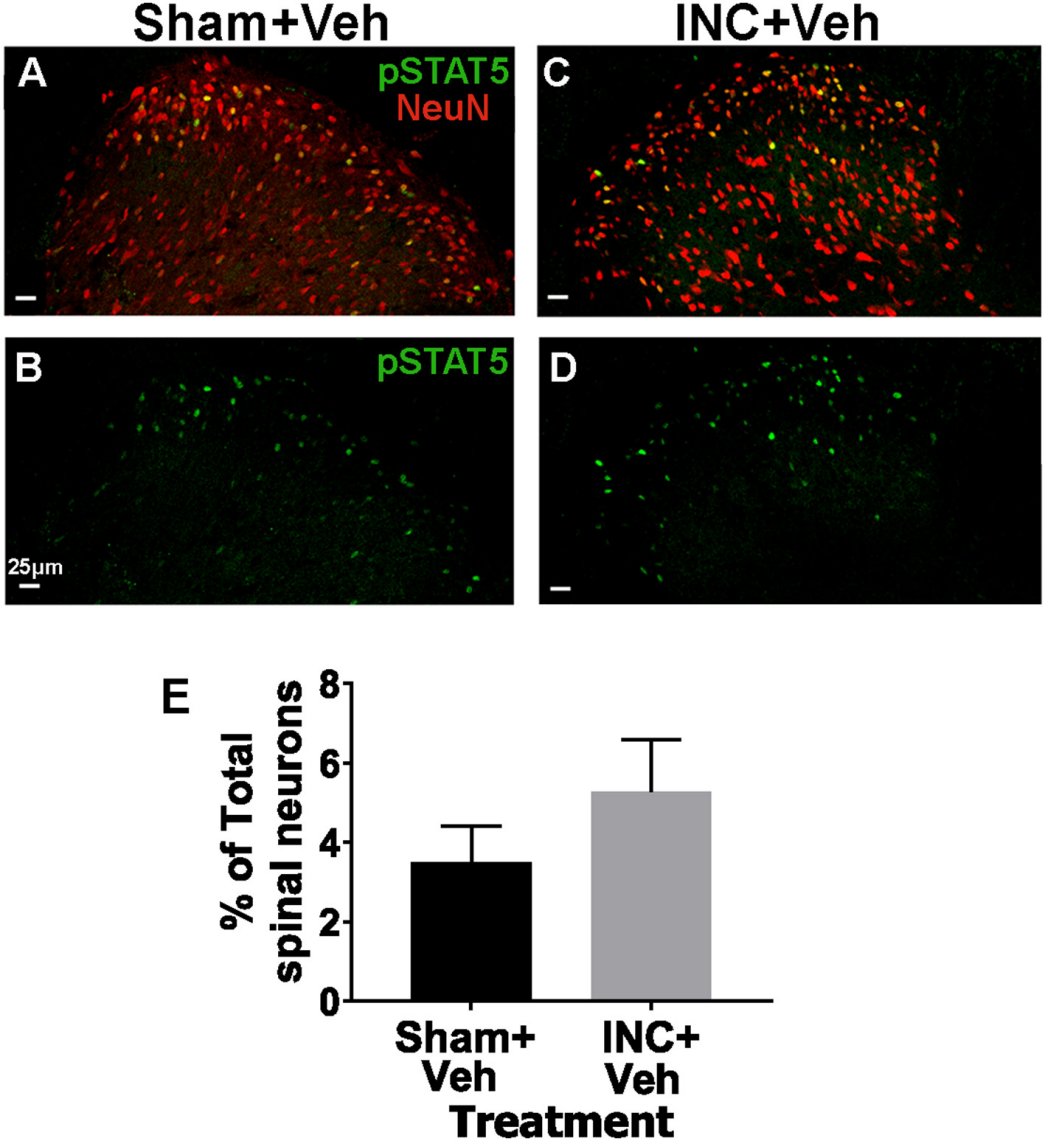
Effect of incision surgery on phosphorylation of STAT5 (pSTAT5) in spinal cord neurons of female rats.Spinal cord sections from female rats were immunolabeled with pSTAT5 and NeuN. NeuN labeling was used to quantify the total number of neurons in the dorsal horn (1,135 ± 77 neurons; *n* = 18 sections). Representative images show pSTAT5 and NeuN labeling in the ipsilateral dorsal horn (laminae I–V): **(A, B)** Rats treated i.p. with vehicle (Veh) for 3 days and collected 1 day after sham surgery (Sham). **(C, D)** Rats treated with vehicle (i.p. for 3 days) and collected 1 day after incision (INC). **(E)** Quantification of pSTAT5^+^ neurons (percentage of total dorsal horn neurons) in the ipsilateral spinal cord of female rats across the indicated treatment groups.Data are presented as mean ± SEM; NS, not significant (one-way ANOVA; *n* = 3). Fluorescent micrographs were captured using a 20 × objective. Scale bar: 25 μm.

Published single-cell sequencing data indicate that *Prlr* mRNA is expressed at very low levels in a small subset of intrinsic spinal cord neurons in mice (Häring et al., 2018). Analysis of Prlr-cre/TdTomato mice showed *Prlr-cre* expression in a subset of lamina I–II spinal neurons in both male and female mice (Patil et al., 2019a). Based on these findings and reports that incision induces PRL_ext_ in the spinal cord (Patil et al., 2013a), we examined whether pSTAT5 is altered in the spinal cord of female rats under postoperative conditions. Basal pSTAT5 labeling was detected in a small subset of lamina I–II spinal neurons (Figures 2A, B). However, incision did not significantly increase pSTAT5^+^ neurons in the ipsilateral dorsal horn (laminae I–V) of female rats (unpaired *t*-test, *t* = 1.1, df = 4, *P* = 0.33, *n* = 3; Figures 2C–E). These results suggest that, unlike DRG neurons, intrinsic spinal cord neuronal PRLR is functionally inactive at postoperative time points, or that the local concentration of incision-induced PRL is insufficient to activate PRLR signaling in dorsal horn neurons. Taken together, incision-induced pSTAT5 in female rat DRG, but not in spinal cord neurons, is likely mediated by PRL_ext_.

### 3.2 Expression of PRL_*ext*_ in sham- and incision-operated rat and mouse tissues

**Figure 3:**
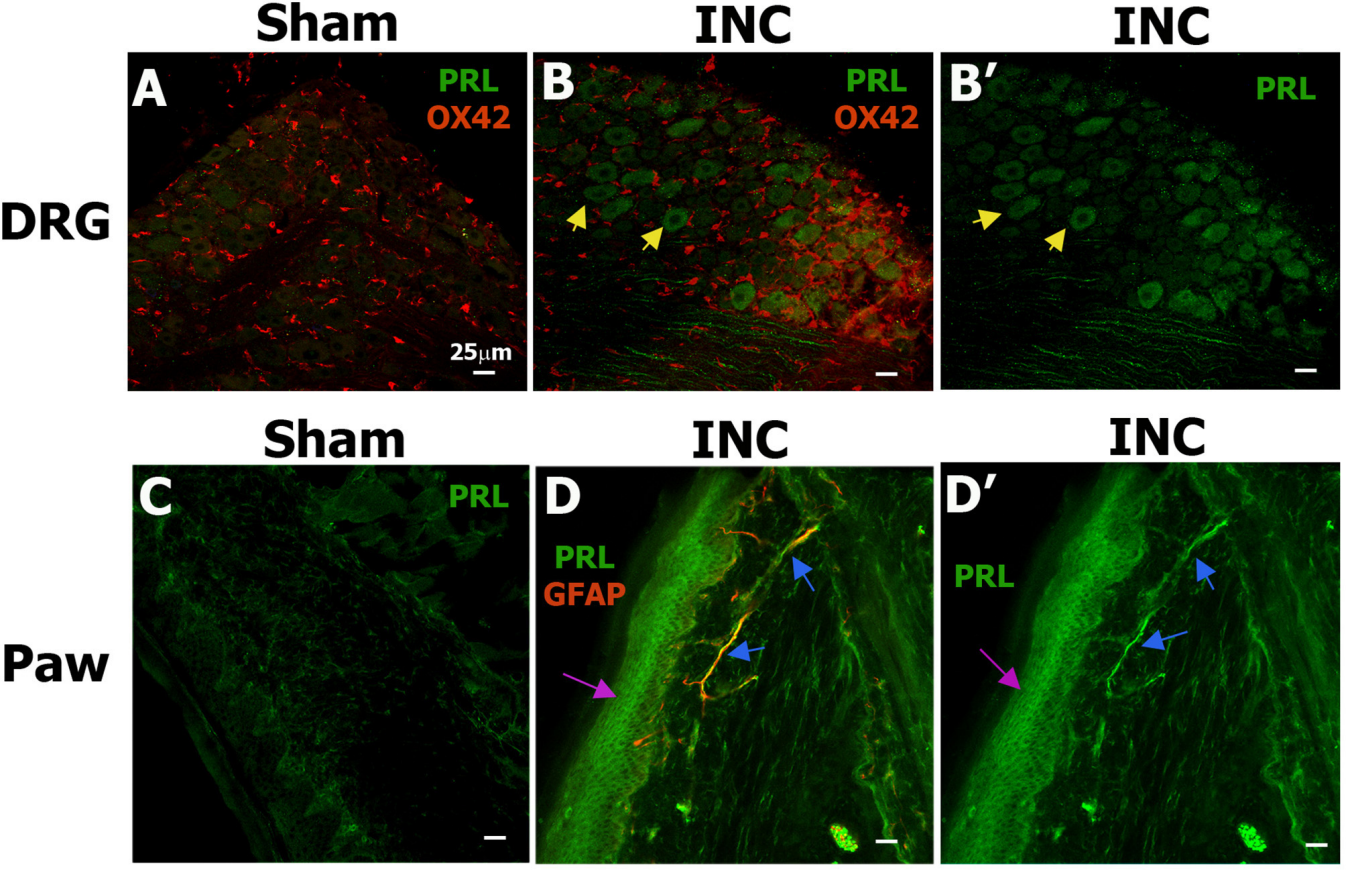
PRL expression in the DRG and hind paw of sham- and incision-operated female rats. **(A–B′)** Immunohistochemical labeling of PRL protein in ipsilateral L4–L5 DRG from female rats 1 day after sham surgery **(A)** or incision **(B, B′)**. OX42 (CD11b/c) was used as a marker for monocytes, macrophages, microglia, and dendritic cells. Yellow arrows indicate PRL^+^ DRG neurons observed after incision (INC). **(C–D′)** PRL labeling in ipsilateral hind paws (Paw) 1 day after sham **(C)** or incision surgery **(D, D′)**. GFAP was used as a glial marker. Blue arrows indicate PRL^+^ afferent myelinated (GFAP^+^) fibers in the dermal layer, and pink arrows indicate PRL^+^ epidermal cells in the hind paw after incision.Tissue type (DRG and paw) and surgical procedures (Sham or INC) are indicated in row and column titles. Images were captured with a 20 × objective. Scale bars: 25 μm for **(A–D)**.

Using IHC, we next examined which cell types in female rat peripheral tissues (hind paw and DRG) and spinal cord produce PRL_ext_ after incision. In sham-operated rats, PRL was undetectable in L5 DRG by antibody labeling (Figure 3A), and single-cell sequencing has confirmed minimal PRL mRNA in DRG neurons (Usoskin et al., 2015; Sharma et al., 2020; Bhuiyan et al., 2024). Incision induced PRL expression in most medium- (30–40 μm) and large-sized (>40 μm) DRG neurons (yellow arrows; 34.5 ± 2.6% of total DRG neurons, *n* = 3), but not in non-neuronal cells, including OX-42^+^ monocytes/macrophages/dendritic cells (Figures 3B, B′) (Mecklenburg et al., 2023). In the hind paw, PRL expression was low in dermal layer cells of sham-operated rats (Figure 3C). Incision triggered PRL expression in myelinated afferent fibers, identified by GFAP^+^ Schwann cells (blue arrows) (Hovhannisyan et al., 2023), and in epidermal keratinocytes (pink arrow; Figures 3D, D′). Previous studies reported PRL_ext_ elevation after surgery but did not identify the cell type (Patil et al., 2013a). Overall, incision induces PRL_ext_ in medium-to-large DRG neurons and epidermal keratinocytes in female rats.

**Figure 4:**
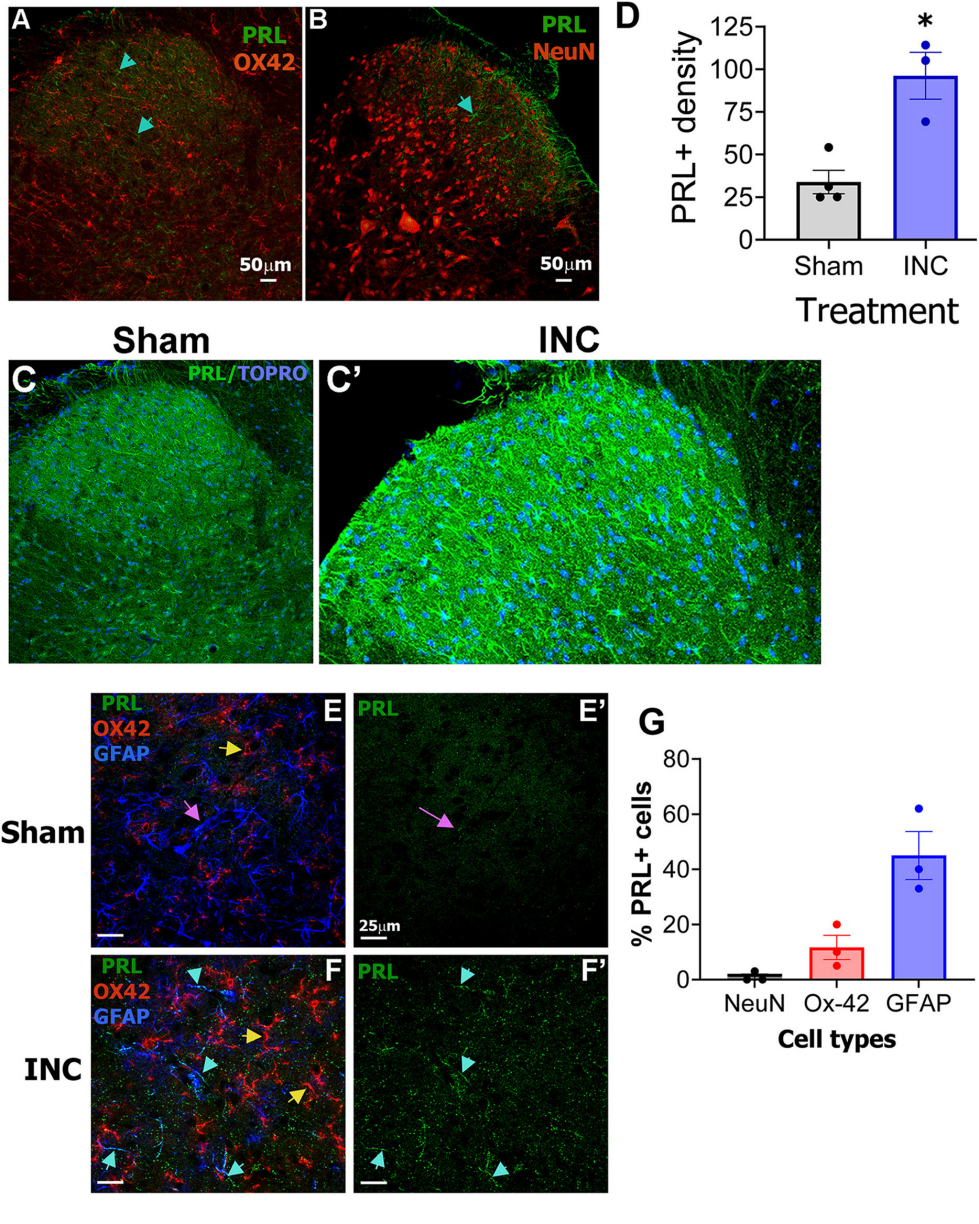
PRL expression in the spinal cord of sham- and incision-operated female rats and mice. **(A, B)** PRL/OX-42 **(A)** and PRL/NeuN co-labeling in the ipsilateral spinal cord (SC) of female rats 1 day after sham. Cyan arrows mark PRL^+^ astrocyte-like cells. NeuN was used as a pan-neuronal marker. Scale bars: 50 μm. **(C, C′)** PRL labeling in SC 1 day after sham and incision (INC) procedure. Images show dorsal horn regions (laminae I–V; partial ventral laminae). Images were captured with a 10 × objective **(A, B, C, C′)**. **(D)** PRL^+^ density in the ipsilateral dorsal horn of SC 1 day after sham and incision (INC) procedure. **(E, E′)** Immunolabeling of PRL in laminae I–II of the ipsilateral spinal cord (SC) from female mice 1 day after sham surgery (Sham). Yellow arrow indicates OX42^+^ microglia, and pink arrows indicate PRL^−^/GFAP^+^ astrocytes. **(F, F′)** PRL labeling in laminae I–II of the ipsilateral SC from female mice 1 day after incision (INC). Yellow arrow indicates OX42^+^ microglia, and cyan arrows indicate PRL^+^/GFAP^+^ astrocytes. Surgical procedures are indicated in row titles. Antibodies and corresponding fluorescence colors are shown in the photomicrographs. Images were captured with a 40 × objective. Scale bar: 25 μm for all panels **(E-F′)**. **(G)** Percentages of PRL^+^ cells in NeuN^+^, Ox-42^+^ and GFAP^+^ in female mice 1 days after INC.

In the spinal cord dorsal horn (laminae I–V) of female rats, PRL immunoreactivity was detected in a subset of cells in sham-operated animals. These PRL^+^ cells were either not detected or found at a minimal level in microglia (OX-42^+^) or neurons (NeuN^+^), and their morphology resembled astrocytes (cyan arrows; Figures 4A, B). Incision changed PRL^+^ intensity in the ipsilateral part of SC dorsal horn of rat females (Figures 4C, C′). ImageJ analyses of PRL^+^ fluorescence intensity revealed a substantial increase after incision (Sham: 33.9 ± 6.9 vs. INC: 96.2 ± 13.7; unpaired *t*-test, *t* = 4.058, df = 3.014, *P* = 0.0267, *n* = 3-4; Figure 4D). In summary, incision induces PRL_ext_ in spinal astrocyte-like cells in female rats.

We next examined whether PRL is also induced by incision surgery in female mouse spinal cord cells. Unlike female rats, PRL levels were very low in the spinal cord of sham-operated female mice (Figures 4E, E′). However, incision on the hind paw induced PRL labeling intensity in a subset of GFAP^+^ spinal astrocytes (Sham: 11.4 ± 3.3 vs. INC: 45.1 ± 5.6; unpaired *t*-test, *t* = 5.2 df = 4, *P* = 0.0066, *n* = 3; Figures 4E′**vs**. 4F′). PRL induction occurred mainly in GFAP^+^ astrocytes in laminae I–V, but few OX42^+^ microglia exhibited PRL^+^ signals (Figures 4F, F′, cyan vs. yellow arrows). Quantification of PRL^+^ cells in female rat NeuN^+^ SC cells as well as female mouse GFAP^+^ and Ox-42^+^ SC cells are shown on the Figure 4G.

**Figure 5:**
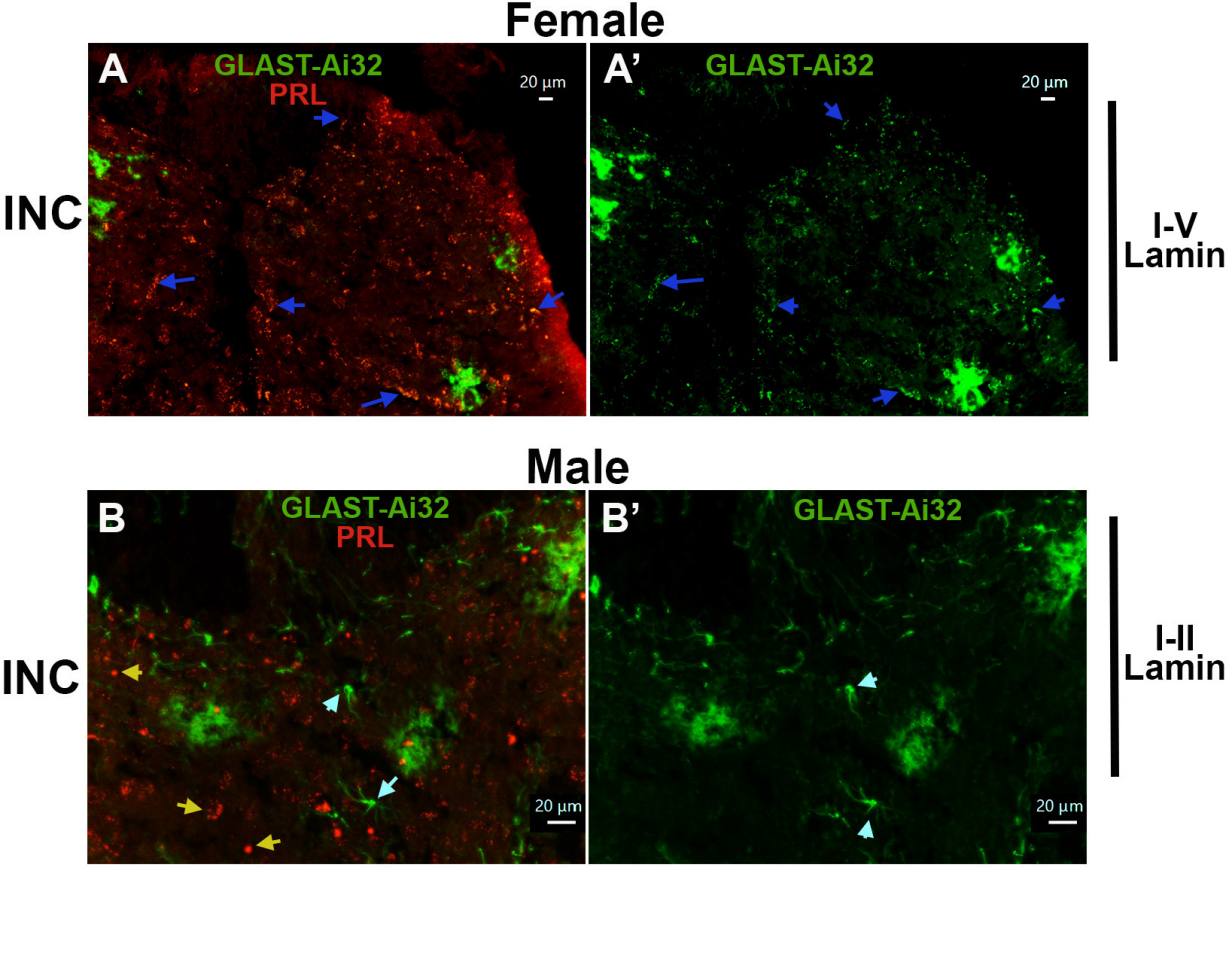
PRL expression in GLAST^+^ spinal astrocytes of incision-operated male and female mice. **(A, A′)** Immunolabeling of PRL in laminae I–V of the spinal cord (SC) from female GLAST^cre−ER/−^/Ai32^fl/−^ 1 day after incision (INC). Blue arrows indicate PRL^+^/GLAST^+^ astrocytes. Images were captured using a 20 × objective. **(B, B′)** PRL labeling in laminae I–II of the SC from male GLAST^cre−ER/−^/Ai32^fl/−^ reporter mice 1 day after incision (INC). Yellow arrows indicate PRL^+^/GLAST^+^ spinal cells, and cyan arrows indicate PRL^+^/GLAST^+^ spinal astrocytes. Experimental procedures and sex of animals are indicated in the figure row titles. Antibodies and corresponding fluorescence colors are shown in the photomicrographs. Images were captured using a 20 × objective for **(A, A′)** and a 40 × objective for **(B, B′)**. Scale bar: 20 μm for all panels.

Besides GFAP, molecular markers commonly used to identify astrocytes in SC are S100B calcium-binding protein, aldehyde dehydrogenase 1 family member L1 (Aldh1L1), and astroglial glutamate transporters such as GLAST (glutamate aspartate transporter, also known as excitatory amino acid transporter 1, EAAT1) and GLT-1 (glutamate transporter 1, also known as EAAT2) (Augusto-Oliveira et al., 2020). GLAST labels 50-70% astrocytes in the adult spinal cord (Rossi, 2015). Since PRL is induced in a subset of astrocytes, we investigated its relationship to GLAST, a marker for a subclass of spinal astrocytes (Rossi, 2015). Using GLAST^cre−ER^/Ai32 (ChR2/YFP) reporter mice, we found that incision-induced PRL co-localized with GLAST^+^ astrocytes in female mice (Figures 5A, A′). In female mice, nearly all GLAST^+^ cells exhibited PRL fluorescence (blue arrows), although only 48 ± 11% of total PRL^+^ cells were GLAST^+^ (*n* = 3; Figures 5A, A′). In contrast, incision did not significantly induce PRL in male SC GLAST^+^ astrocytes (Sham: 7.8 ± 1.5 vs. INC: 12.3 ± 1.6; unpaired *t*-test, *t* = 2.0, df = 4, *P* = 0.1155, *n* = 3; Figures 5B, B′). Furthermore, many PRL^+^ cells in males were GLAST-Ai32 negative (yellow vs. cyan arrows; Figures 5B, B′). Together, these results indicate that incision-induced PRL in a majority of GLAST^+^ spinal astrocytes in female rodents.

### 3.3 Contribution of PRL_*pit*_ to incision and inflammation-induced hypersensitivity in female rats and mice

**Figure 6:**
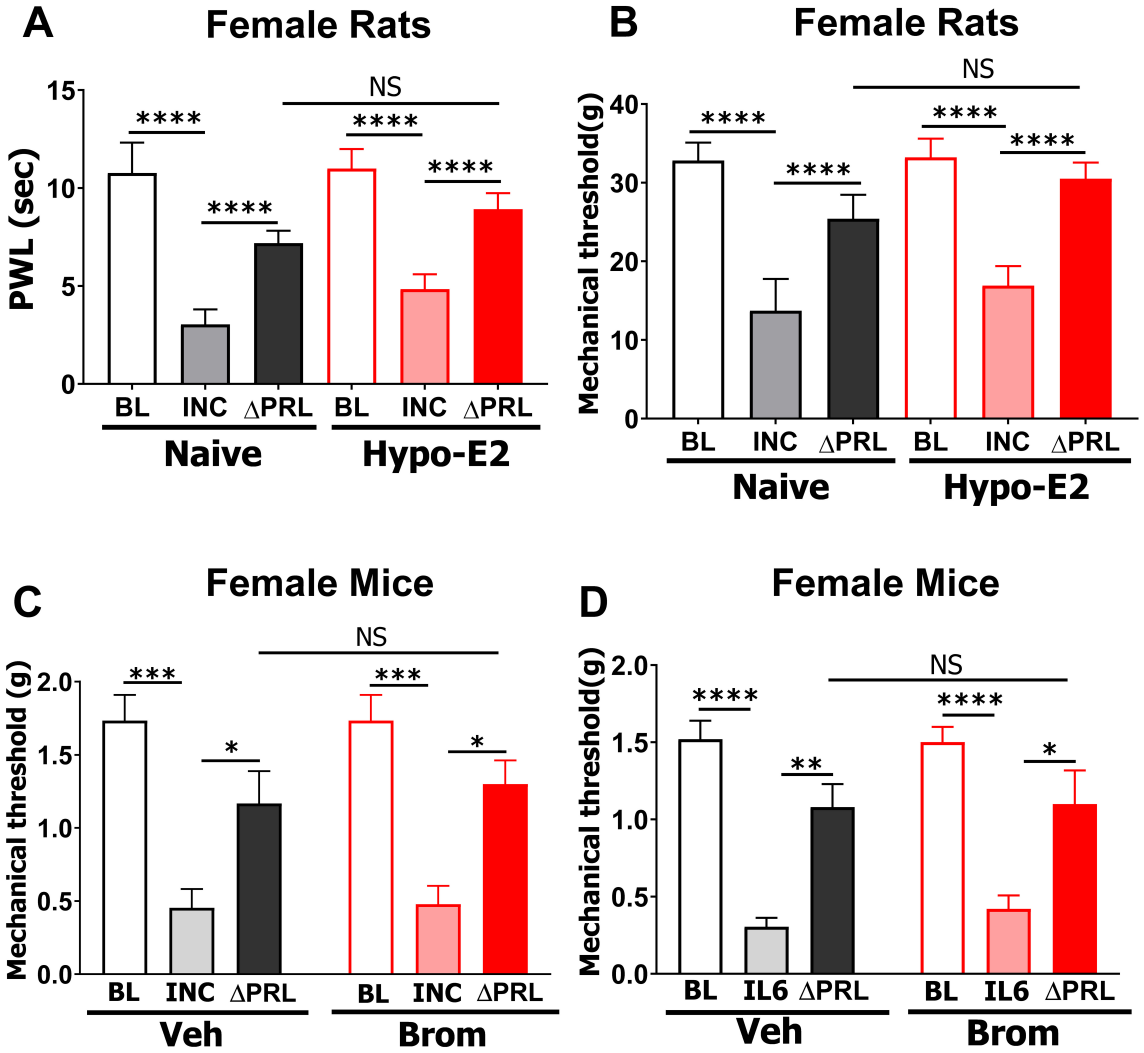
Effect of pituitary-derived PRL (PRL_pit_) on postoperative and inflammatory pain in female rats and mice. **(A, B)** Naïve and hypophysectomized female rats supplemented with estradiol (Hypo-E2) underwent incision surgery (INC). The PRLR antagonist ΔPRL (5 μg) was administered i.t. 1 day post-incision. Heat **(A)** and mechanical **(B)** hypersensitivity in ipsilateral hind paws were evaluated using the Dynamic Plantar Aesthesiometer at baseline (BL), 1 day post-incision (INC), and 1 hour after ΔPRL injection (ΔPRL). Statistical analysis: two-way ANOVA with Bonferroni's *post hoc* test (NS, *p* > 0.05; **** *p* < 0.0001; *n* = 6). **(C, D)** Naïve female mice were treated i.p. with vehicle (Veh) or bromocriptine (Brom) for 3 consecutive days. BL mechanical thresholds were measured using von Frey filaments. Mechanical hypersensitivity in ipsilateral hind paws was induced by incision (INC; **C**) or by intraplantar injection of 1 ng IL-6 (IL-6; **D**). One day post-incision or IL-6 injection, ΔPRL (5 μg, i.t.) was administered, and mechanical hypersensitivity was assessed 1 h later (ΔPRL). Statistical analysis: two-way ANOVA with Bonferroni's *post hoc* test (NS, *p* > 0.05; *p < 0.05; **p < 0.01; ***p < 0.001; **** *p* < 0.0001; *n* = 6).

Production and release of PRL_pit_ can be effectively blocked (or substantially suppressed) by systemic bromocriptine mesylate treatment (Sapsford et al., 2012; Mason et al., 2022) or surgical removal of the pituitary (i.e., hypophysectomy) (Emanuele et al., 1986). Female hypophysectomized rats were supplemented with estradiol (Hypo-E2), because hypophysectomy significantly lowers circulating gonadal hormones, including estrogen, which is essential for PRLR function in sensory neurons (Anderson and Chatterton, 1976; Patil et al., 2019a; Paige et al., 2020; Avona et al., 2021). Consistent with previous reports (Green et al., 2016), baseline nociception and 1-day post-incision heat and mechanical hypersensitivity in the ipsilateral hind paw were not altered in Hypo-E2 compared to naïve female rats (Figures 6A, B). Importantly, blocking spinal PRLR with intrathecal ΔPRL (5 μg) produced a comparable reduction in thermal and mechanical hypersensitivity in both naïve and Hypo-E2 rats. The interaction *F* ratio between naïve and Hypo-E2 groups after ΔPRL treatment was not statistically significant [2-way ANOVA; heat: *F*_(2, 24)_ = 2.1, *P* = 0.14; mechanical: *F*_(2, 24)_ = 1.8, *P* = 0.19; *n* = 6; Figures 6A, B].

Next, we assessed whether PRL_pit_ is essential for hypersensitivity in acute pain models in female mice (Patil et al., 2019a). Systemic bromocriptine treatment to suppress PRL_pit_ in female mice did not alter baseline nociception or incision- (INC) or IL-6-induced mechanical hypersensitivity in the ipsilateral hind paw (Figures 6C, D). Spinal administration of ΔPRL produced a comparable reduction in mechanical hypersensitivity in both vehicle- and bromocriptine-treated female mice in the postoperative model [2-way ANOVA; *F*_(2, 30)_ = 0.09, *P* = 0.91, *n* = 6; Figure 6C] and the IL-6 inflammatory pain model [2-way ANOVA; *F*_(2, 27)_ = 0.13, *P* = 0.88, *n* = 6; Figure 6D]. Together, these results indicate that the previously reported female-selective role of peripheral PRLR signaling in acute pain (Patil et al., 2019a) is mainly independent of PRL_pit_ in both female rats and mice.

### 3.4 Role of GLAST^+^ spinal cells and PRL_*ext*_ in incision-induced pain hypersensitivity in mice

Although specific ablation of PRL in astrocytes is currently not feasible (a PRL^fl/fl^ mouse line is not available), an alternative approach is to ablate astrocytes in adult mice using a DTA^fl/fl^ mouse line crossed with an astrocyte-specific Cre reporter line (Brockschnieder et al., 2006a,b). In this strategy, Cre recombinase can be driven in astrocytes by GFAP, GLAST, or glutamate transporter 1 (GLT-1) promoters (Mobley and Mccarty, 2012). However, GLAST, GLT-1, and GFAP are also expressed in embryonic glial precursor cells (Domowicz et al., 2011). GFAP is further expressed in satellite glial cells (Stephenson and Byers, 1995) and Schwann cells (Mokuno et al., 1989). To selectively eliminate ${\text{PRL}}_{\text{ext}}^{+}$ astrocytes in the CNS while avoiding deletion of astrocyte precursors and other non-astrocyte glial cells, we used the inducible GLAST^cre−ER/−^ mouse line. GLAST^+^ cells were ablated by tamoxifen treatment of GLAST^cre−ER/−^/DTA^fl/−^ postnatal mice, while DTA^fl/−^ mice served as controls. Behavioral experiments were conducted 4–6 weeks after the last tamoxifen injection.

**Figure 7:**
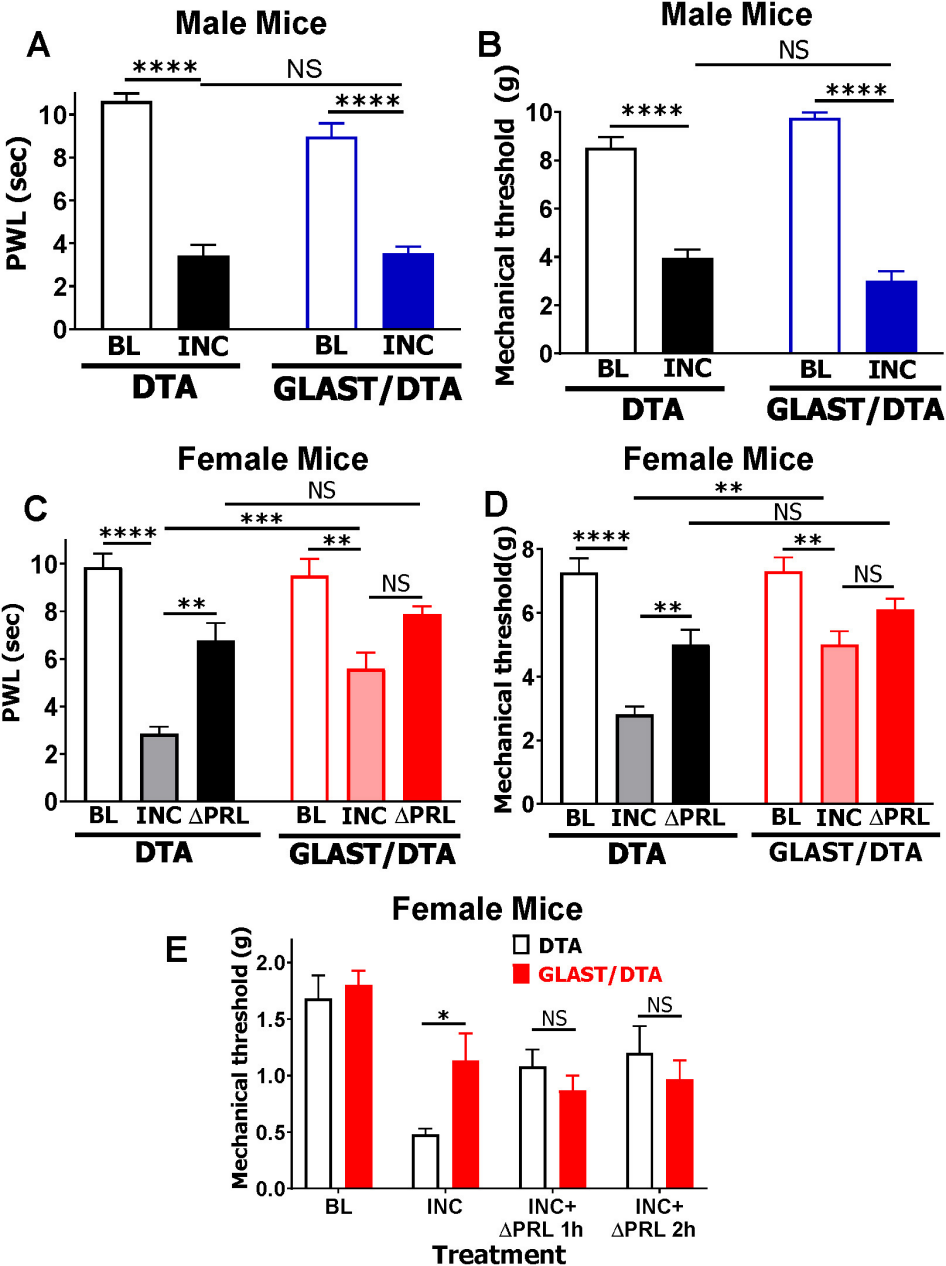
Role of extra-pituitary PRL (PRL_est_) from GLAST^+^ spinal cells in postoperative pain in male and female mice. **(A, B)** Male DTA^fl/−^ (DTA) and GLAST^cre−ER/−^/DTA^fl/−^ (GLAST/DTA) mice underwent incision surgery (INC). Heat **(A)** and mechanical **(B)** hypersensitivity in ipsilateral hind paws were assessed 1 day post-incision. **(C, D)** Female DTA and GLAST/DTA mice underwent INC. One day post-incision, ΔPRL (5 μg) was administered intrathecally (i.t.). Heat **(C)** and mechanical **(D)** hypersensitivity in ipsilateral hind paws were measured at 1 day post-INC and 1 H after ΔPRL treatment (ΔPRL). **(E)** Female DTA and GLAST/DTA mice were treated i.p. with bromocriptine for 3 consecutive days before undergoing INC. One day post-incision, ΔPRL (5 μg, i.t.) was injected, and mechanical hypersensitivity was measured 1 day post-INC, and 1 and 2 h post-ΔPRL (INC + 1h ΔPRL, INC + 2h ΔPRL). Mechanical hypersensitivity **(B, D)** was measured using the Dynamic Plantar Aesthesiometer, and in **(E)** using von Frey filaments (up-down method). Baseline (BL) heat and mechanical nociception were measured prior to surgery. PWL, paw withdrawal latency. Statistical analysis: two-way ANOVA with Bonferroni's *post hoc* test (NS, *p* > 0.05; *p < 0.05; **p < 0.01; ***p < 0.001; ****p < 0.0001; *n* = 4-6).

In male mice, ablation of GLAST^+^ cells did not alter incision-induced heat [2-way ANOVA; *F*_(1, 14)_ = 3.9; *P* = 0.07; *n* = 4–5; Figure 7A] or mechanical hypersensitivity [2-way ANOVA; *F*_(1, 14)_ = 8.7; *P* = 0.11; *n* = 4–5; Figure 7B] in the ipsilateral hind paw, consistent with the low levels of PRL_ext_ upregulation and its absence in GLAST^+^ cells in male spinal cord (Figures 5B, 5B′). In female mice, however, ablation of GLAST^+^ cells partially reversed incision-induced heat [2-way ANOVA; *F*_(2, 24)_ = 3.4; *P* = 0.048; *n* = 6; Figure 7C] and mechanical hypersensitivity [2-way ANOVA; *F*_(2, 24)_ = 3.6; *P* = 0.042; *n* = 6; Figure 7D] in the ipsilateral hind paw. Additionally, intrathecal (i.t.) ΔPRL (5 μg) in control female mice significantly reduced thermal (Figure 7C) and mechanical (Figure 7D) hypersensitivity. In contrast, ΔPRL had no additional effect in female mice lacking GLAST^+^ cells (Figures 7C, D). To rule out contributions of PRL_pit_, GLAST^cre−ER/−^/DTA^fl/−^ and DTA^fl/−^ female mice were pre-treated with bromocriptine. ΔPRL remained effective in DTA controls but had no additional effect in GLAST^+^ cell-ablated mice (Figure 7E). These results indicate that PRL_ext_ from GLAST^+^ astrocytes is a primary driver of sex-dependent hypersensitivity, while PRL_pit_ contributes negligibly to incision- or inflammation-induced hypersensitivity in female rodents.

## 4 Discussion

Peripheral, spinal, or dural administration of exogenous PRL induces transient but robust hypersensitivity in a female-specific manner (Patil et al., 2019a; Avona et al., 2021). This effect arises from sex-selective differences in PRLR and *Prlr* mRNA expressions in female DRG CGRP^+^ nociceptors, whereas PRLR and *Prlr* mRNA levels are minimal in DRG and trigeminal ganglion (TG) CGRP^+^ neurons of males (Patil et al., 2019a; Avona et al., 2021). Consequently, PRL selectively activates sensory neurons in females but not males (Diogenes et al., 2006; Belugin et al., 2013; Avona et al., 2021). Blocking PRLR signaling in the periphery (hind paw and dura) or spinal cord markedly and selectively reverses incision-, inflammation-, and CGRP-induced hypersensitivity in females (Patil et al., 2019a; Avona et al., 2021). This study examined source of endogenous PRL, which activated PRLR in postoperative and inflammatory pain conditions. It demonstrated that under conditions of tissue injury and acute inflammation, the primary endogenous source of PRL contributing to female-selective hypersensitivity is extra-pituitary PRL (PRL_ext_) supplied by a subset of dorsal horn astrocytes.

Under normal physiological conditions, circulating levels of endogenous PRL are low to moderate (< 20 ng/ml) and are derived almost exclusively from the pituitary gland (Freeman et al., 2000). An exception occurs during late pregnancy and lactation, when PRL_pit_ increases dramatically, reaching hyperprolactinemic levels exceeding 100 ng/ml (Yip et al., 2019). Various pathological conditions associated with endometriosis, tissue injury or stress in both rodents and humans have been shown to elevate serum PRL through increased pituitary production and release (Berczi et al., 1984; Emanuele et al., 1986; Noreng et al., 1987; Mateo et al., 1998; Yardeni et al., 2007; Scotland et al., 2011; Patil et al., 2013a; Kirk et al., 2017; Ikegami et al., 2022; Mason et al., 2022; Kopruszinski et al., 2023; Lee et al., 2025; Stratton et al., 2025). However, these elevations are typically modest compared to those observed during pregnancy or lactation, with serum PRL levels rising to approximately 20–60 ng/ml (Jaroenporn et al., 2007; Patil et al., 2013a; Kirk et al., 2017; Patil et al., 2019a; Yip et al., 2019; Ikegami et al., 2022). Under pathological or sensitized conditions, tissue injury or stress may enhance peripheral and/or spinal PRLR signaling within trigeminal and spinal systems, allowing even moderate elevations of PRL_pit_ to contribute to neuronal hypersensitivity (Patil et al., 2019a; Avona et al., 2021; Mason et al., 2022; Stratton et al., 2025).

PRL_pit_ has been shown to sensitize sensory neurons in trigeminal pain conditions such as endometriosis, headache and migraine, including stress-induced preclinical models (Chen et al., 2020a; Ikegami et al., 2022; Mason et al., 2022; Kopruszinski et al., 2023; Stratton et al., 2025). However, our findings indicate that PRL_pit_ contributes minimally to tissue injury- and acute inflammation-induced hypersensitivity in the trunk region of female rats and mice (Figure 6). This suggests that the primary mediator of PRLR signaling driving female-selective postoperative and acute inflammatory pain in the trunk regions, such as hind paw, is likely PRL_ext_.

Substantial expression of PRL in extra-pituitary tissues in naive conditions is well documented in humans and, to a lesser extent, in rodents (Benjonathan et al., 1996; Featherstone et al., 2012). A defining feature of human PRL_ext_ is its transcription from an alternative promoter not present in rodents (Berwaer et al., 1994; Gerlo et al., 2006). Despite this difference, PRL_ext_ mRNA and protein have been identified in various rodent non-neuronal and neuronal cell types under naive and especially pathological conditions (Benjonathan et al., 1996; Marano and Ben-Jonathan, 2014). PRL_ext_ expression has been reported in mammary tissue during breast cancer (Shaw-Bruha et al., 1997), prostate epithelial and smooth muscle cells (Nevalainen et al., 1997), vascular endothelial cells (Clapp et al., 1998; Ochoa et al., 2001), immune cells such as granulocytes and *T*-cells (Wu et al., 1996; Xu et al., 2010), skin keratinocytes (Goldstein et al., 2014), adipocytes (Hugo et al., 2008; Scotland et al., 2011), glial cells (Möderscheim et al., 2007; Rivera et al., 2008); and neurons (Mejia et al., 1997; Diogenes et al., 2006). Although PRL_ext_ has been proposed to play physiological and pathological roles, direct evidence were remained limited (Featherstone et al., 2012; Christensen et al., 2013).

This study tested hypothesis that PRL_ext_ could play a vital pathophysiological role during certain conditions. Literature and our data indicate that PRL_ext_ protein can be detected after tissue damage and acute inflammation in DRG neurons, and certain non-neuronal cell types of skin and spinal cord. Thus, it is known that PRL_ext_ levels increase after inflammation or tissue injury in the hind paw and spinal cord of rodents (Scotland et al., 2011; Patil et al., 2013a,b). These elevations are slightly higher in female hind paw tissues and markedly greater in the spinal cord, where PRL_ext_ upregulation occurs selectively in females following surgery or inflammation (Scotland et al., 2011; Patil et al., 2013a,b). Provided here data extend these findings, showing that hind paw incision in female rats robustly induces PRL_ext_ in myelinated afferent fibers and epidermal cells of the hind paw, as well as in medium-to-large DRG neurons and spinal astrocytes (Figures 3, 4). Similarly, in female mice, PRL_ext_ was induced in a subset of spinal astrocytes, some of which were GLAST-positive (Figure 5). These findings also indicate that there are a potential common sex-dependent mechanism after mild tissue injury and acute inflammation.

pSTAT5 as a surrogate marker for PRL responsiveness is localized with neurons (NeuN^+^) in the spinal cord (Figure 2), whereas PRL itself does not appear to be present in NeuN cells in the SC (Figure 3). Since pSTAT5 is downstream target of PRLR signaling, PRL as the ligand probably comes from non-neuronal spinal cells and acts via a paracrine, but not autocrine mechanisms, to target intrinsic spinal neurons and/or central terminals of sensory neuronal fibers both of which express PRLR and *Prlr* mRNA (Patil et al., 2019a,b). In contrast, in DRG, TG and dura mater, PRL signaling could potentially use both paracrine and autocrine mechanisms (Stephenson and Byers, 1995; Patil et al., 2019a; Avona et al., 2021; Mason et al., 2022; Mei et al., 2025).

Next, we examined the functional significance of PRL_ext_. Hind paw incision produces a transient (< 2 days) and mild (≈25–50%) increase in GFAP intensity in the superficial dorsal horn of the spinal cord in male rats (Obata et al., 2006; Liu et al., 2015) and mice (Romero et al., 2013). Other studies indicate that p38 activation during acute postoperative pain occurs in microglia, not astrocytes (Huang et al., 2011), and astrocyte activation in acute (< 7 days) postoperative pain in female rodents has not been reported. In male rats, intrathecal administration of fluorocitrate (1 nmol), a selective inhibitor of the astrocytic Krebs cycle (Hassel et al., 1992), at 1 day post-incision reduces hypersensitivity (Obata et al., 2006). Our data demonstrate ablation of GLAST^+^ astrocytes reverses both acute postoperative and inflammatory hypersensitivity in female mice (Figures 7C–E), but not in males (Figures 7A, B). This effect likely reflects the contribution of astrocytic PRL_ext_, which is induced by incision and expressed in GLAST^+^ spinal astrocytes in females but not males (Figure 5), and is one of key mediators of female-specific postoperative and acute inflammatory pain (Figures 7A–D). In contrast, PRL_pit_ plays a minimal role in this mechanism (Figure 7E). Importantly, these findings are likely specific to GLAST^+^ astrocytes; ablation of PRL_ext_-expressing astrocytes using GFAP^cre−ER^ reporter mice could yield different results, as GFAP is also expressed in satellite glial cells (Stephenson and Byers, 1995) and Schwann cells (Mokuno et al., 1989), many of which do not express PRL (Patil et al., 2019b). Consistent with previous reports (Huang et al., 2011; Romero et al., 2013), our data confirm that astrocytes contribute little to acute pain in males. Several studies suggest that GFAP^+^ spinal cells are involved in the maintenance of persistent pain rather than in acute pain states (Tanga et al., 2004; Romero et al., 2013; Zhang et al., 2018). Here, we show for the first time that spinal astrocytes mediate acute postoperative pain in female, but not male mice via PRL_ext_. The role of PRL_ext_ in chronic pain and the maintenance of persistent pain remains unknown, as sustained induction of PRL_ext_ following stress, tissue, or nerve injury has not yet been investigated.

In conclusion, our study demonstrates that tissue injury elevates not only PRL_pit_ (Patil et al., 2013a), but also PRL_ext_ in both neuronal and non-neuronal cells of the hind paw, DRG, and spinal cord in female rodents. Importantly, this increase in PRL_ext_ is sex-dependent, and PRL_ext_ expressed in GLAST^+^ spinal astrocytes mediates female-selective acute postoperative and inflammatory pain hypersensitivity. Thus, GLAST^+^ astrocytes are critical contributors to tissue injury- and acute inflammation-induced hypersensitivity in females, but not males. We propose that spinal PRL_ext_ functions as a neuromodulator by activating PRLR on central sensory neuronal terminals and/or intrinsic PRLR^+^ spinal neurons, sensitizing and modulating Ca^2+^ (including TRPV1 and TRPA1) and K^+^ channels, and enhancing neurotransmission from central terminals of sensory neurons to spinal dorsal horn neurons and/or from spinal neurons to other CNS regions involved in pain pathway (Patil et al., 2019a). These findings provide a new perspective on sex differences in pain signaling and reinforce the pivotal role of the PRL system, specifically PRLR and PRL_ext_, including glial PRL_ext_, in setting nociceptive thresholds in females.

## Data Availability

The raw data supporting the conclusions of this article will be made available by the authors, without undue reservation.
